# Benign Emptying After Right Lower Lobectomy Mimicking a Bronchopleural Fistula

**DOI:** 10.7759/cureus.100366

**Published:** 2025-12-29

**Authors:** Akihito Gunji, Tomonari Oki, Shuhei Iizuka, Toru Nakamura

**Affiliations:** 1 Department of General Thoracic Surgery, Seirei Hamamatsu General Hospital, Hamamatsu, JPN

**Keywords:** bronchopleural fistula, lobectomy, pneumonectomy, postoperative period, pulmonary surgical procedures

## Abstract

Bronchopleural fistula (BPF) is a serious and potentially fatal postoperative complication after pulmonary resection. Benign emptying of the postpneumonectomy space (BEPS) is a rare, self-limiting condition characterized by a sudden reduction in pleural fluid volume without evidence of fistula formation, observed after pneumonectomy. To date, BEPS-like conditions following lobectomy have not been reported. Because the clinical and radiological manifestations of BEPS overlap significantly with those of BPF, distinguishing between them is challenging. We present an unusual case of a BEPS-like phenomenon that developed after a right lower lobectomy and initially mimicked a BPF both clinically and radiographically.

A 77-year-old man with a history of interstitial pneumonia and prostate cancer presented with right lower lobe adenocarcinoma. He underwent video-assisted thoracoscopic right lower lobectomy with lymph node dissection and was discharged without complications. Five weeks after surgery, he developed increased sputum production and mild dyspnea. Chest radiography revealed a reduction in the right pleural effusion, raising a strong suspicion of BPF. Exploratory thoracoscopy showed a well-healed bronchial stump without air leakage on saline irrigation or indocyanine green dye testing. These results supported the diagnosis of a BEPS-like condition. The patient’s symptoms resolved spontaneously without therapeutic intervention, and follow-up imaging confirmed stable lung expansion and no recurrence at five months after exploratory thoracoscopy.

This case highlights that a self-limiting BEPS-like phenomenon may occur even after lobectomy and can closely mimic BPF in presentation. Because the consequences of overlooking BPF are life-threatening, a cautious diagnostic approach integrating clinical evaluation, imaging, bronchoscopy, and, when indicated, surgical exploration is imperative before adopting conservative management. Awareness of this condition is crucial for thoracic surgeons to avoid unnecessary invasive interventions while ensuring prompt management of true BPF when present.

## Introduction

Bronchopleural fistula (BPF) is a serious and potentially life-threatening complication following pulmonary resection. The reported incidence of BPF ranges from approximately 4.5% to 20% after pneumonectomy and 0.5% to 1.0% after lobectomy [1,2], with higher rates observed after right pneumonectomy and particularly after right lower lobectomy [3]. Known risk factors include a heavy smoking history, poor nutritional status, bronchial ischemia, and use of corticosteroids [4]. Due to the risk of severe complications such as empyema or even bronchial-pulmonary arterial fistula, early diagnosis and prompt surgical intervention-often including open-window thoracostomy-are frequently required [5,6]. Benign emptying of the postpneumonectomy space (BEPS) is a rare, self-limiting phenomenon characterized by a sudden decrease in pleural fluid without evidence of a BPF [7]. BEPS is observed after pneumonectomy and is thought to result from transient pleural fluid absorption or pressure equilibration rather than actual air leakage. To date, BEPS-like phenomena after lobectomy have not been reported, and differentiating them from true BPF can be challenging because of overlapping clinical and radiologic findings. We herein report a rare case of a BEPS-like phenomenon that occurred after right lower lobectomy, mimicking BPF.

## Case presentation

A 77-year-old man presented with an infiltrative opacity in the right lower lobe on chest radiography. His past medical history was notable for interstitial pneumonia and prostate cancer (cT2bN1M1c). He was initially treated with a gonadotropin-releasing hormone (GnRH) agonist and an androgen synthesis enzyme inhibitor five years ago, which continued for two years. Subsequently, he underwent surgical castration, after which both agents were discontinued. Three years later, an increase in serum prostate-specific antigen (PSA) levels was observed, prompting a switch to an anti-androgen agent, which has continued to the present.

**Figure 1:**
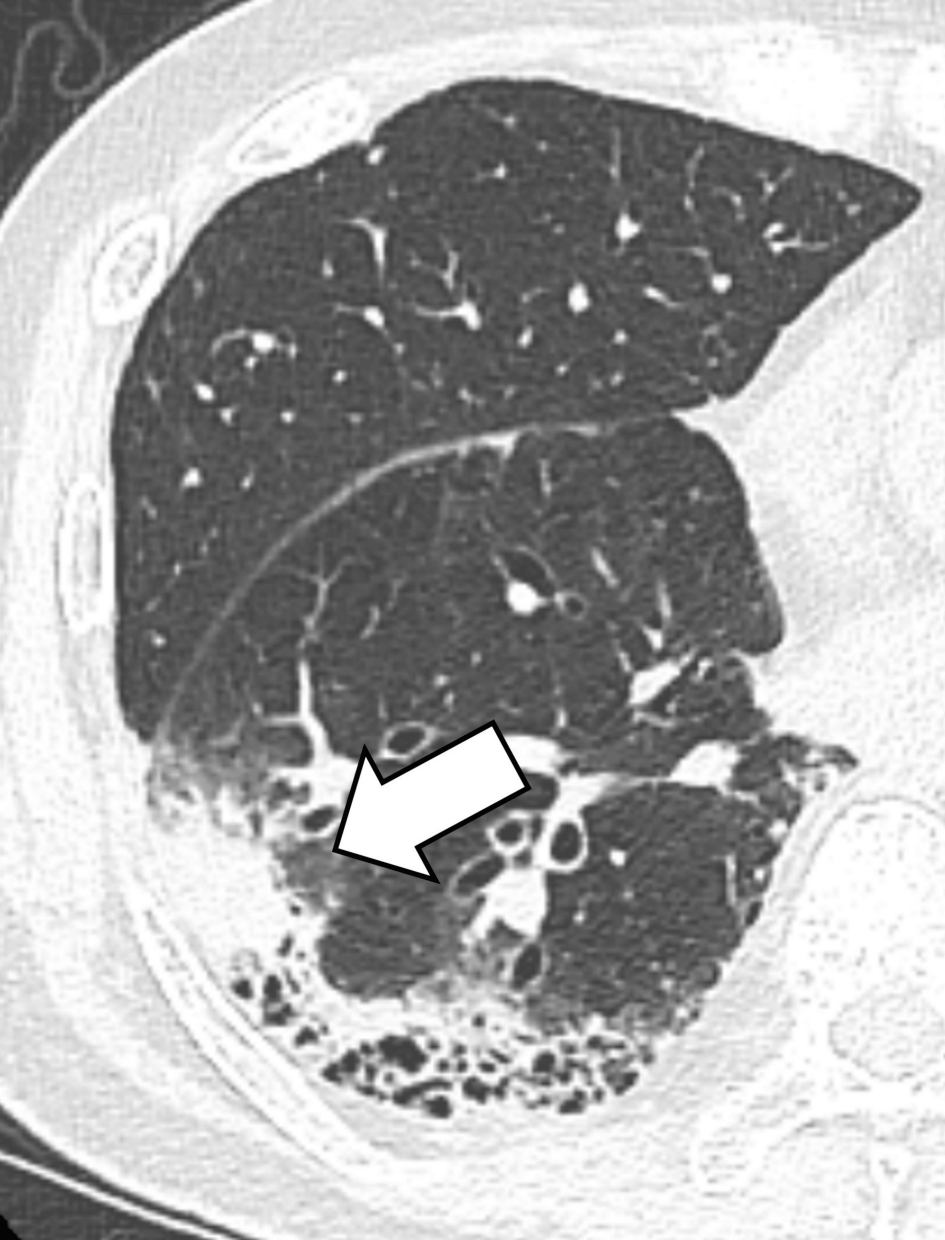
Chest CT before surgery Contrast-enhanced CT scan showing a consolidation with honeycomb cystic changes in the right lower lobe (arrow). CT: computed tomography

**Figure 2:**
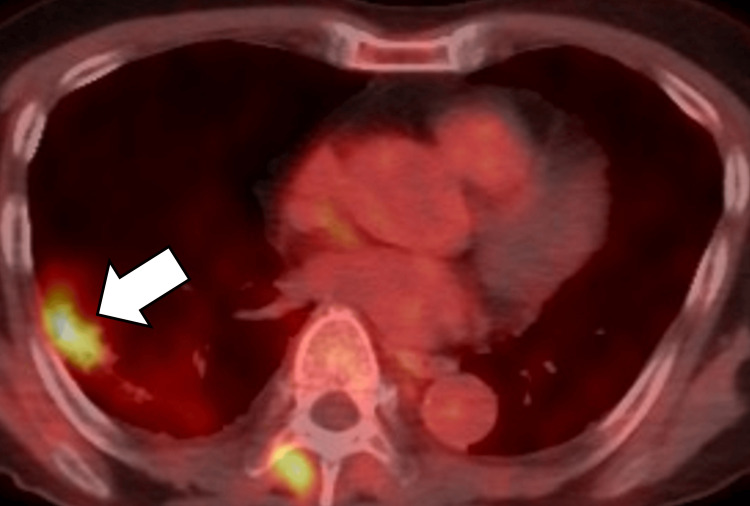
PET-CT before surgery PET-CT scan showing increased fluorodeoxyglucose uptake in the consolidation with honeycomb cystic changes in the right lower lobe (arrow). PET-CT: positron emission tomography-computed tomography

He had a smoking history of 20 cigarettes per day from age 18 to 60. Laboratory testing revealed tumor marker levels within normal ranges (carcinoembryonic antigen (CEA) 3.90, cytokeratin 19 fragment (CYFRA) <1.0, pro-gastrin-releasing peptide (Pro-GRP) 45.9, and Sialyl Lewis X (SLX) 29). He had no history of diabetes mellitus. Contrast-enhanced computed tomography (CT) revealed an 88 mm infiltrative lesion with honeycomb-like cystic changes in the right lower lobe (Figure 1). On positron emission tomography (PET)-CT, this lesion showed fluorodeoxyglucose (FDG) uptake with a maximum standardized uptake value (SUVmax) of 7.58. Transbronchial biopsy confirmed lung adenocarcinoma (Figure 2).

The patient was diagnosed with right lower lobe adenocarcinoma (cT4N0M0, stage IIIA) and underwent video-assisted thoracic right lower lobectomy with ND1b lymph node dissection. No bronchial stump reinforcement with muscle or pericardial fat was performed. He was discharged on postoperative day (POD) 5. No adjuvant chemotherapy was administered, and he was followed as an outpatient.

**Figure 3:**
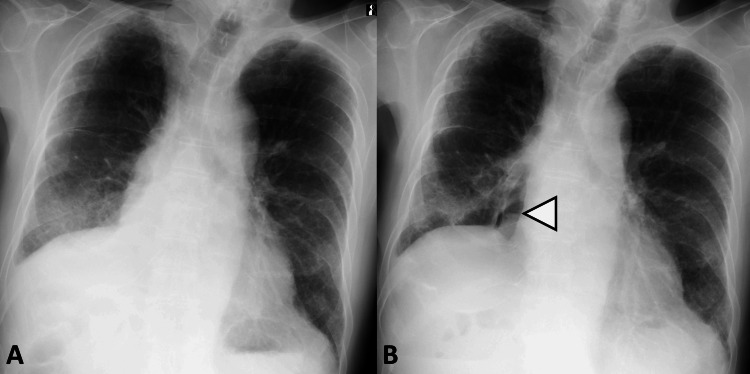
Chest radiographs after right lower lobectomy (A) Chest radiograph obtained at the second outpatient visit one month after discharge showing excellent lung expansion. (B) Chest radiograph one week later showing the air-fluid level and hyperlucency in the right lower lung field (arrowhead).

**Figure 4:**
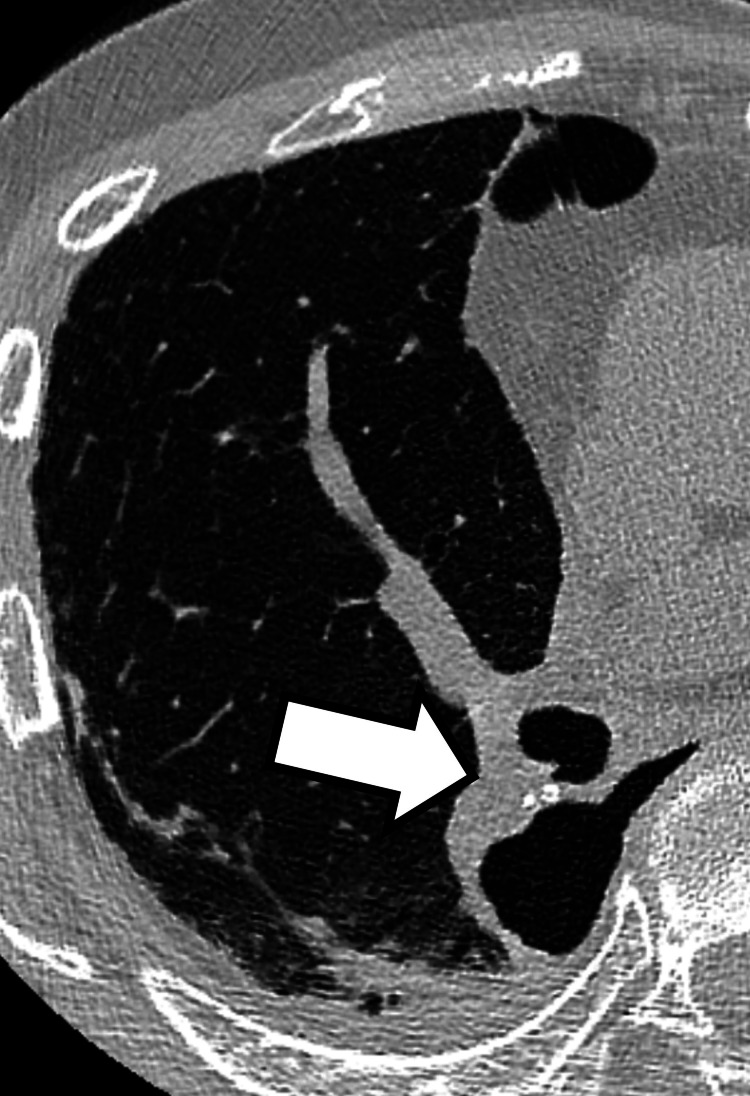
Chest CT before exploratory thoracoscopy CT image showing a pneumothorax without an obvious bronchial fistula (arrow), and no pleural effusion was observed. CT: computed tomography

At the outpatient follow-up visit one month after discharge, he reported general fatigue but denied fever, hemoptysis, or increased sputum volume. Chest radiography at that time demonstrated good lung expansion, consistent with the expected postoperative appearance following right lower lobectomy (Figure 3A). One week later, he developed increased sputum production and dyspnea. Chest radiography revealed a reduction in the right pleural effusion (Figure 3B). The simultaneous increase in watery sputum and decrease in pleural effusion raised suspicion of a bronchial stump fistula rather than an alveolar-pleural fistula [8,9]. Although chest CT revealed no evident fistula formation (Figure 4), exploratory thoracoscopy was performed under general anesthesia because BPF was still suspected. Preoperative bronchoscopy was omitted because CT demonstrated no definitive findings.

**Figure 5:**
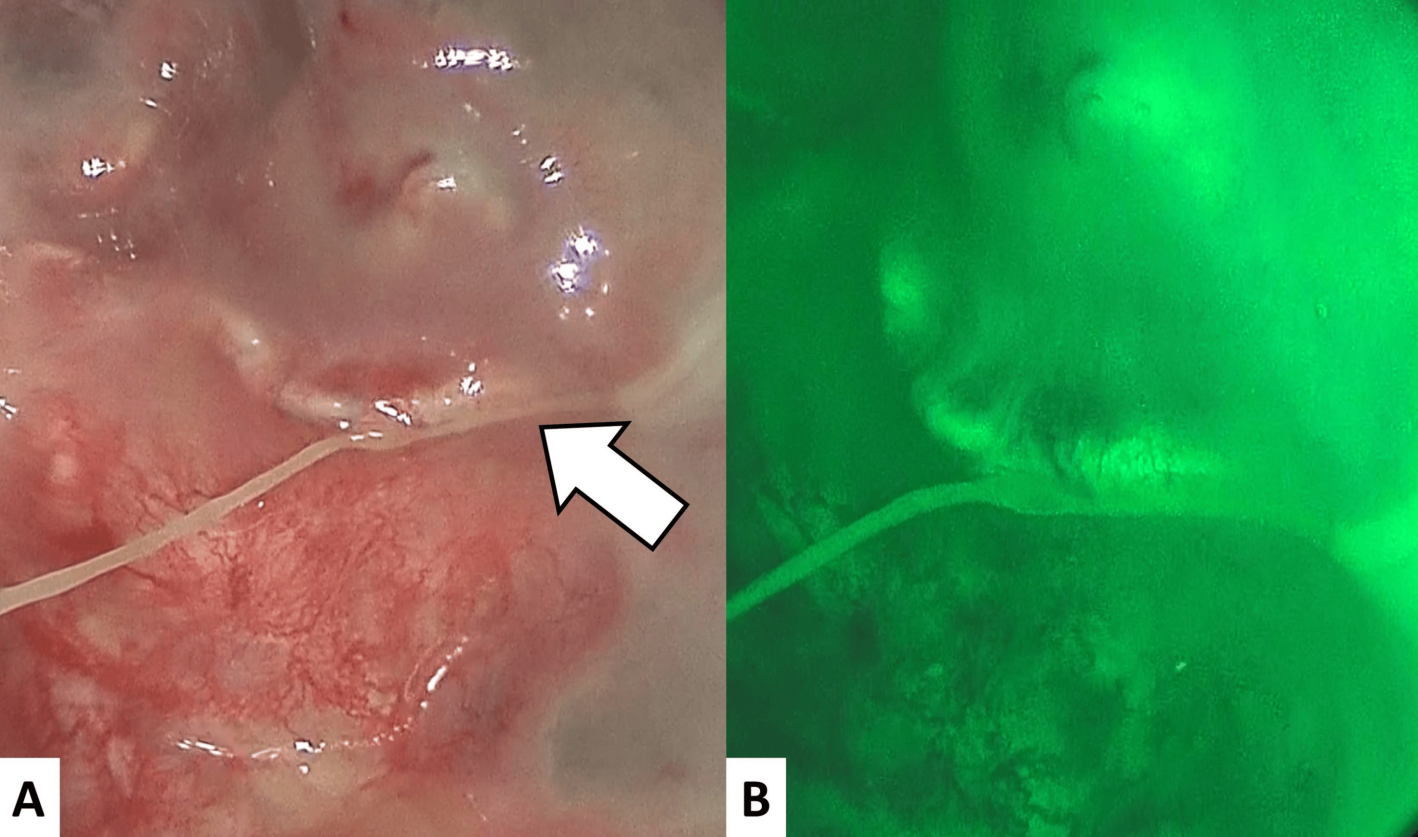
Operative view (A) Thoracoscopic observation showed the bronchial stump covered with epithelial tissue and no fistula formation (arrow). (B) While the thoracic cavity was being filled with an indocyanine green solution, bronchoscopic observation revealed no evidence of dye leakage into the bronchial lumen.

Thoracoscopic findings revealed that the lung surface, bronchial stump, and vascular stumps were all well epithelialized without fistula formation (Figure 5A).

After irrigation of the thoracic cavity, an air leak test under positive pressure revealed no air leakage from the bronchial stump or lung parenchyma. Subsequently, indocyanine green diluted in saline was instilled into the pleural cavity (Figure 5B). While suction was applied to induce physiological negative pressure in the airway under bronchoscopic observation, no dye leakage into the bronchial lumen was detected. These findings excluded BPF and were instead consistent with a BEPS-like condition.

The patient’s symptoms gradually resolved without the development of empyema. At five months after exploratory thoracoscopy, the right lung remained well expanded, with adequate pleural fluid retention and no signs of infection.

## Discussion

This case highlights two important clinical insights. First, a clinical condition mimicking BEPS can occur following lobectomy. Second, even when a BEPS-like process is suspected, a high index of suspicion for BPF must be maintained, as overlooking this more severe condition can result in fatal consequences.

Both BPF and BEPS represent postoperative intrathoracic changes and are reported to occur more frequently on the right side [3]. Although differentiation between these two entities can be clinically challenging, it is crucial given their markedly different management strategies and prognoses.

Alveolar-pleural fistulas-typically a milder condition that often resolves spontaneously-are characterized by air leakage without aspiration of pleural effusion into the tracheobronchial tree [9]. In the present case, the rapid reduction in pleural effusion accompanied by a marked increase in watery sputum made the presence of alveolar-pleural fistula unlikely.

BEPS is a rare postoperative complication, occurring in approximately 0.29%-0.65% of pneumonectomy cases. It is characterized by a sudden reduction in the pleural fluid level on imaging, forming an air-containing cavity without evidence of infection or BPF [7,10]. Several mechanisms have been proposed to explain the development of BEPS. One hypothesis involves an occult BFS, where the fistula permits air passage but is too narrow to allow bacterial migration, thus remaining sterile. Other proposed mechanisms include congenital or iatrogenic diaphragmatic defects permitting communication between the pleural and peritoneal cavities. Fluid leaks into the surrounding soft tissues through the chest wall. Despite these hypotheses, the exact etiology remains unclear [7].

The diagnosis of BEPS is primarily clinical and based on exclusion. Diagnostic features typically include the absence of fever and leukocytosis, no increase in sputum production, no evidence of fistula on bronchoscopy, and negative pleural fluid cultures. Importantly, BEPS is self-limiting and usually resolves without medical or surgical intervention, highlighting the importance of accurate recognition to avoid overtreatment.

In contrast, BPF often necessitates prompt diagnostic and therapeutic measures because it can be immediately fatal [11,12]. Diagnostic modalities include bronchoscopic balloon occlusion testing, methylene blue administration via chest drain or airway, and high-resolution or multidetector CT imaging to visualize fistula tracts. However, a negative bronchoscopic or radiological study does not completely exclude the diagnosis of BPF [13].

In the present case, several findings were suggestive of BPF, including the use of corticosteroids, a right lower lobectomy (a procedure known to carry a higher BPF risk), and a sudden decrease in pleural effusion accompanied by an increase in sputum production. In contrast, the absence of fever, together with normal inflammatory markers, was inconsistent with the typical presentation of BPF. Consequently, negative findings on thoracoscopy ruled out the diagnosis of a BEPS-like condition.

Delayed diagnosis of BPF can lead to catastrophic consequences, such as a broncho-pulmonary artery fistula [11,12,14,15]. In this case, early diagnosis was particularly crucial because the patient had undergone right lower lobectomy, which carries an increased risk of BPF, and was additionally immunodeficient due to prostate cancer and preoperative steroid use.

Exploratory thoracoscopy was chosen with consideration including open-window thoracostomy in case of BPF instead of conservative observation or preceding bronchoscopic evaluation [16,17]. In retrospect, this approach may have been excessively invasive. However, given the higher likelihood and severity of BPF, immediate exclusion of the diagnosis was mandatory.

In summary, even when postoperative pleural changes suggest self-limiting BEPS, clinicians must remain alert for the more likely and life-threatening possibility of BPF. Comprehensive evaluation, including the clinical course, imaging, bronchoscopy, culture results, and, when necessary, surgical exploration, is essential before conservative management can be safely adopted.

## Conclusions

A condition mimicking BEPS may occur even after a lobectomy. Because its clinical and radiological findings closely resemble those of BPF, a careful differential diagnosis is indispensable. Given the life-threatening nature of BPF, a comprehensive diagnostic evaluation, including assessment of the clinical course, imaging studies, and, when necessary, exploratory thoracoscopy, should be undertaken to reliably exclude this condition. Even when a BEPS-like condition is suspected, a passive watchful waiting strategy is not advisable; instead, cautious and proactive management is warranted.
